# Supplementary material for: Association between maternal mental health and early childhood development, nutrition, and common childhood illnesses in Khwisero subcounty, Kenya
Source: PLoS One. 2025 Jan 17;20(1):e0317762. doi: 10.1371/journal.pone.0317762 (PMC11741660; doi:10.1371/journal.pone.0317762)
Supplement: S1 File — (PDF) [file pone.0317762.s001.pdf]

amref health africa

Amref Health Africa in Kenya

REF: AMREF – ESRC P1060-2021 **October 17, 2022**

Amanuel Abajobir
African Population and Health Research Center
P.O. Box 10787-00100
Nairobi, Kenya
Tel: +254 20 400 1000
Email: aabajobir@aphrc.org

Dear Amanuel Abajobir,

RESEARCH PROTOCOL: EXAMINING THE IMPACT OF MATERNAL MENTAL HEALTH ON CHILD HEALTH OUTCOMES AND THE ROLE OF INNOVATIVE PARTNERSHIP FOR UNIVERSAL AND SUSTAINABLE HEALTHCARE (I-PUSH) INTERVENTION: A CLUSTER RANDOMISED CONTROLLED TRIAL STUDY.

SECONDARY TITLE: EXAMINING THE EFFECT OF COVID-19 ON MATERNAL MENTAL HEALTH

Thank you for submitting your protocol to the Amref Ethics and Scientific Review Committee (ESRC).

This is to inform you that the ESRC has reviewed and approved the annual renewal of your protocol with the following amendments.

1. Amendment of the term *"perinatal depression"* in the primary title to *"mental health"* and removal of the term *"the roles of pregnancy and i-PUSH intervention"* in the secondary title because of limited number of pregnant women in the follow-up.
2. Protocol modifications that reflect changes regarding the replacement of perinatal depression with mental health and removal of the component on roles of pregnancy and i-PUSH intervention.

The approval period is from October 17, 2022, to November 7, 2023, and is subject to compliance with the following requirements:

a) Only approved documents (including informed consents, study instruments, advertising materials, material transfer agreements etc.) will be used.
b) All changes including (amendments, deviations, violations etc.) are submitted for review and approval by Amref ESRC before implementation.
c) Death and life-threatening problems and severe adverse events (SAEs) or unexpected adverse events whether related or unrelated to the study must be reported to the Amref ESRC within 72 hours of notification.
d) Any changes, anticipated or otherwise that may increase the risks or affect safety or welfare of study participants and others or affect the integrity of the research must be reported to Amref ESRC within 72 hours.
e) Clearance for export of biological specimen must be obtained from the relevant government authorities for each batch of shipment/export.
f) Submission of a request for renewal of approval at least 60 days prior to expiry of the approval period. Attach a comprehensive progress report to support the renewal.
g) In case of late renewal, the Amref ESRC shall not be held responsible for any severe adverse events (SAEs) that may occur as a result of research activities that were carried out after the expiry of approval.
h) Submission of an executive summary report within 90 days upon completion of the study to the Amref ESRC.

Board Members: **Mr P Kasimu | Mrs E Mathu | Prof P Kiama | Mrs M Kuyoh | Prof Z Qureshi | Prof J Wang'ombe | Dr D Soti | Dr G Gitahi**

Winner of the Gates Award for Global Health
BILL & MELINDA GATES FOUNDATION

i) All government regulations for prevention and control of the spread of COVID-19 including social distancing, provision of personal protective equipment for participants and research assistants should be adhered to during data collection. All research assistants should be monitored for COVID 19 symptoms and referred for testing in case they present with symptoms.

Prior to commencing your study, you will be expected to obtain a research license from National Commission for Science, Technology and Innovation (NACOSTI) https://research-portal.nacosti.go.ke and also obtain other clearances needed.

Please do not hesitate to contact the ESRC Secretariat (esrc.kenya@amref.org) for any clarification or query.

Yours sincerely,

AMREF - ESRC
12 OCT 2022
P.O. BOX 30125 - 00100, NAIROBI

Prof. Mohamed Karama
Chair, Amref ESRC
CC: Samuel Muhula, Monitoring & Evaluation and Research Manager, Amref Health Africa in Kenya.

Board Members: Mr P Kasimu | Mrs E Mathu | Prof P Kiama | Mrs M Kuyoh | Prof Z Qureshi | Prof J Wang'ombe | Dr D Soti | Dr G Gitahi

P O Box 30125-00100 Nairobi, Tel: +254 (0)20 699 4000, Fax: +254 (0)20 699 2531. www.amref.org

Winner of the Gates Award for Global Health
BILL & MELINDA GATES FOUNDATION
