# Supplementary material for: Association between maternal mental health and early childhood development, nutrition, and common childhood illnesses in Khwisero subcounty, Kenya
Source: PLoS One. 2025 Jan 17;20(1):e0317762. doi: 10.1371/journal.pone.0317762 (PMC11741660; doi:10.1371/journal.pone.0317762)
Supplement: S3 File — (DOCX) [file pone.0317762.s003.docx]

| **Supplementary Table 1.** Factors associated with ECD | | | | | | | |
| --- | --- | --- | --- | --- | --- | --- | --- |
|  | **Unadjusted** | | | | **Adjusted** | | |
| **Characteristic** |  | **POR** | **95%CI** | **p-value** | **POR** | **95%CI** | **p-value** |
| **Cognitive** |  |  |  |  |  |  |  |
| Sex of child |  |  |  |  |  |  |  |
| Female |  |  | — |  |  | — |  |
| Male |  | 1.43 | 0.09, 2.03 | 0.30 | 1.42 | 0.13, 1.84 | 0.14 |
| Age of child (years) |  | 3.32 | 2.98, 3.66 | <0.001 | 3.34 | 2.94, 3.75 | <0.001 |
| Maternal age (years) |  |  |  |  |  |  |  |
| 15 – 24 |  |  | — |  |  | — |  |
| 25 – 24 |  | 3.58 | 0.56, 23.00 | 0.20 | 1.29 | 0.07, 1.26 | 0.10 |
| 35 - 44 |  | 4.31 | 0.49, 13.47 | 0.20 | 1.11 | 1.01, 5.00 | 0.01 |
| 45 - 49 |  | 1.10 | 0.00, 8.85 | 0.90 | 1.08 | 0.00, 7.66 | 0.30 |
|  |  |  |  |  |  |  |  |
| Maternal educational status |  |  |  |  |  |  |  |
| Primary not completed |  |  | — |  |  | — |  |
| At least primary |  | 1.43 | 0.26, 7.85 | 0.70 | 1.86 | 0.25, 2.94 | 0.80 |
| Maternal literacy |  |  |  |  |  |  |  |
| Cannot read and write |  |  | — |  |  | — |  |
| Can read and write |  | 5.58 | 0.14, 7.89 | 0.40 | 7.39 | 0.29, 9.31 | 0.20 |
| Mothers’ income |  |  |  |  |  |  |  |
| Not own income |  |  | — |  |  | — |  |
| Own income |  | 3.46 | 1.83, 5.32 | 0.001 | 1.98 | 1.17, 13.46 | 0.02 |
| Mothers’ marital status |  |  |  |  |  |  |  |
| Never married |  |  | — |  |  | — |  |
| Married |  | 3.25 | 0.15, 18.90 | 0.50 | 4.19 | 0.23, 9.01 | 0.30 |
| Cohabiting |  | 1.86 | 0.02, 15.53 | 0.80 | 4.83 | 0.12, 13.89 | 0.40 |
| Divorced/separated |  | 8.87 | 0.72, 23.65 | 0.60 | 6.01 | 0.00, 27.92 | 0.40 |
| Widowed |  | 7.91 | 0.00, 19.86 | 0.30 | 2.14 | 0.00, 17.08 | 0.80 |
| Maternal history of any chronic disease |  |  |  |  |  |  |  |
| No |  |  | — |  |  | — |  |
| Yes |  | 1.98 | 0.11, 8.46 | 0.90 | 1.52 | 0.10, 5.80 | 0.40 |
| Presence of father in the household |  |  |  |  |  |  |  |
| Father lived in same household |  |  | — |  |  | — |  |
| Father was not a household member and was living in the same community |  | 1.21 | 0.67, 7.53 | 0.06 | 1.67 | 0.00, 7.99 | 0.60 |
| Father was not a household member and was not living in the same community |  | 1.54 | 0.04, 12.01 | 0.07 | 2.06 | 0.13, 11.27 | 0.80 |
| Father was dead |  | 1.32 | 0.76, 5.33 | 0.11 | 2.69 | 0.00, 7.26 | 0.80 |
| Household wealth terciles |  |  |  |  |  |  |  |
| Poor |  |  | — |  |  | — |  |
| Middle |  | 1.01 | 0.03, 3.64 | 0.90 | 1.43 | 0.03, 5.34 | 0.50 |
| Rich |  | 1.47 | 0.02, 4.12 | 0.70 | 1.30 | 0.02, 3.85 | 0.40 |
| Decision to child health-seeking |  |  |  |  |  |  |  |
| Mother/caregiver alone |  |  | — |  |  | — |  |
| Husband/partner/head alone |  | 1.09 | 1.01, 1.82 | 0.034 | 1.22 | 0.04, 1.59 | 0.07 |
| Husband/partner/head together |  | 1.55 | 0.09, 3.49 | 0.50 | 1.82 | 0.21, 3.32 | 0.80 |
| Another member in the household |  | 1.10 | 0.00, 1.94 | 0.60 | 1.00 | 0.00, 9.71 | 0.70 |
| Mother/caregiver and another member in the household |  | 1.00 | 0.00, 1.22 | 0.06 | 1.17 | 0.00, 2.89 | 0.80 |
| Paid care |  |  |  |  |  |  |  |
| No |  |  | — |  |  | — |  |
| Yes |  | 1.86 | 1.09, 7.33 | <0.001 | 2.95 | 0.39, 12.64 | 0.30 |
| Language and general communication |  |  |  |  |  |  |  |
| Sex of child |  |  |  |  |  |  |  |
| Female |  |  | — |  |  | — |  |
| Male |  | 0.05 | 0.00, 19.19 | 0.20 | 0.89 | 0.00, 7.73 | 0.90 |
| Age of child (years) |  | 3.86 | 1.69, 7.03 | <0.001 | 1.69 | 1.26, 8.12 | <0.001 |
| Maternal age (years) |  |  |  |  |  |  |  |
| 15 - 24 |  |  | — |  |  | — |  |
| 25 - 24 |  | 1.86 | 10.3, 9.43 | 0.04 | 1.91 | 0.60, 4.77 | 0.40 |
| 35 - 44 |  | 1.61 | 1.04, 6.82 | 0.01 | 1.51 | 0.16, 8.14 | 0.60 |
| 45 - 49 |  | 1.15 | 0.02, 4.53 | 0.30 | 1.15 | 0.11, 7.42 | 0.50 |
| Maternal educational status |  |  |  |  |  |  |  |
| Primary not completed |  |  | — |  |  | — |  |
| At least primary |  | 1.26 | 0.47, 9.98 | 0.80 | 0.35 | 0.71, 7.01 | 0.90 |
| Maternal literacy |  |  |  |  |  |  |  |
| Cannot read and write |  |  | — |  |  | — |  |
| Can read and write |  | 1.75 | 0.89, 17.4 | 0.90 | 0.94 | 0.22, 15.34 | 0.70 |
| Mothers’ income |  |  |  |  |  |  |  |
| Not own income |  |  | — |  |  | — |  |
| Own income |  | 2.07 | 0.22, 6.33 | 0.06 | 2.78 | 0.53, 10.09 | 0.5 |
| Household wealth terciles |  |  |  |  |  |  |  |
| Poor |  |  | — |  |  | — |  |
| Middle |  | 1.59 | 0.19, 23.37 | 0.50 | 1.45 | 0.71, 17.6 | 0.80 |
| Rich |  | 2.53 | 0.16, 20.22 | 0.80 | 0.33 | 0.15, 14.92 | 0.90 |
| Mothers’ marital status |  |  |  |  |  |  |  |
| Never married |  |  | — |  |  | — |  |
| Married |  | 1.60 | 0.55, 24.74 | 0.30 | 1.08 | 0.39, 8.24 | 0.30 |
| Cohabiting |  | 0.44 | 0.34, 17.47 | 0.60 | 1.53 | 0.39, 2.32 | 0.08 |
| Divorced/separated |  | 1.00 | 0.18, 6.18 | 0.60 | 2.57 | 0.57, 4.43 | 0.70 |
| Widowed |  | 2.00 | 0.93, 53.93 | 0.20 | 1.09 | 0.52, 7.34 | 0.70 |
| Presence of father in the household |  |  |  |  |  |  |  |
| Father lived in same household |  |  | — |  |  | — |  |
| Father was not a household member and was living in the same community |  | 2.17 | 1.21, 9.12 | 0.004 | 0.64 | 0.91, 2.18 | 0.80 |
| Father was not a household member and was not living in the same community |  | 1.85 | 1.28, 5.72 | <0.001 | 1.82 | 0.89, 4.54 | 0.90 |
| Father was dead |  | 1.36 | 1.16, 3.55 | <0.001 | 1.13 | 0.18, 6.7 | 0.80 |
| Maternal history of any chronic disease |  |  |  |  |  |  |  |
| No |  |  | — |  |  | — |  |
| Yes |  | 3.29 | 0.92, 4.5 | 0.60 | 0.32 | 0.76, 10.4 | 0.90 |
| Decision to child health-seeking |  |  |  |  |  |  |  |
| Mother/caregiver alone |  |  | — |  |  | — |  |
| Husband/partner/head alone |  | 0.00 | 0.00, 0.79 | 0.04 | 0.86 | 0.82, 1.10 | 0.08 |
| Husband/partner/head together |  | 1.59 | 0.92, 3.73 | 0.07 | 1.35 | 0.80, 1.80 | 0.13 |
| Another member in the household |  | 0.00 | 0.00, 0.01 | 0.03 | 1.04 | 0.36, 7.29 | 0.08 |
| Mother/caregiver and another member in the household |  | 0.19 | 0.06, 3.31 | 0.09 | 1.20 | 0.13, -3.28 | 0.09 |
| Paid care |  |  |  |  |  |  |  |
| No |  |  | — |  |  | — |  |
| Yes |  | 1.01 | 0.23, 8.47 | 0.09 | 1.91 | 0.08, 10.26 | 0.80 |
| Self-help/adaptation |  |  |  |  |  |  |  |
| Sex of child |  |  |  |  |  |  |  |
| Female |  |  | — |  |  | — |  |
| Male |  | 1.61 | 0.31, 2.09 | 0.06 | 1.86 | 0.02, 2.30 | 0.14 |
| Age of child (years) |  | 3.69 | 3.33, 4.06 | <0.001 | 3.39 | 2.98, 3.79 | <0.001 |
| Maternal age (years) |  |  |  |  |  |  |  |
| 15 - 24 |  |  | — |  |  | — |  |
| 25 - 24 |  | 3.58 | 1.65, 5.51 | <0.001 | 1.93 | 0.53, 2.39 | 0.20 |
| 35 - 44 |  | 2.51 | 2.25, 6.78 | <0.001 | 0.67 | 0.12, 2.47 | 0.50 |
| 45 - 49 |  | 2.94 | 0.89, 9.77 | 0.40 | 1.93 | 0.66, 5.52 | 0.70 |
| Maternal educational status |  |  |  |  |  |  |  |
| Primary not completed |  |  | — |  |  | — |  |
| At least primary |  | 1.43 | 0.37, 2.23 | 0.60 | 1.05 | 0.19, 1.29 | 0.90 |
| Maternal literacy |  |  |  |  |  |  |  |
| Cannot read and write |  |  | — |  |  | — |  |
| Can read and write |  | 1.75 | 0.18, 4.68 | 0.70 | 1.24 | 0.49, 3.00 | 0.90 |
| Mothers’ income |  |  |  |  |  |  |  |
| Not own income |  |  | — |  |  | — |  |
| Own income |  | 2.01 | 1.32, 3.7 | 0.02 | 1.36 | 0.87, 1.59 | 0.60 |
| Mothers’ marital status |  |  |  |  |  |  |  |
| Never married |  |  | — |  |  | — |  |
| Married |  | 3.46 | 1.20, 6.73 | 0.04 | 1.71 | 0.21, 3.62 | 0.60 |
| Cohabiting |  | 1.05 | 0.69, 4.58 | 0.90 | 1.09 | 0.77, 2.59 | 0.60 |
| Divorced/separated |  | 3.45 | 0.71, 13.6 | 0.50 | 1.29 | 0.14, 9.72 | 0.90 |
| Widowed |  | 4.75 | 0.12, 11.61 | 0.20 | 0.12 | 0.09, 4.84 | 0.70 |
| Maternal history of any chronic disease |  |  |  |  |  |  |  |
| No |  |  | — |  |  | — |  |
| Yes |  | 0.92 | -1.38, 3.22 | 0.40 | -0.03 | -1.73, 1.67 | 0.90 |
| Presence of father in the household |  |  |  |  |  |  |  |
| Father lived in same household |  |  | — |  |  | — |  |
| Father was not a household member and was living in the same community |  | 1.93 | 1.43, 16.44 | 0.003 | 3.39 | 0.27, 11.06 | 0.40 |
| Father was not a household member and was not living in the same community |  | 1.95 | 1.09, 13.8 | <0.001 | 1.43 | 0.43, 8.28 | 0.70 |
| Father was dead |  | 1.76 | 0.83, 15.68 | <0.001 | 2.31 | 0.58, 9.21 | 0.50 |
| Household wealth terciles |  |  |  |  |  |  |  |
| Poor |  |  | — |  |  | — |  |
| Middle |  | 1.32 | 0.34, 4.97 | 0.50 | 1.14 | 0.69, 2.41 | 0.90 |
| Rich |  | 1.35 | 0.28, 3.99 | 0.80 | 1.07 | 0.64, 2.50 | 0.90 |
| Decision to child health-seeking |  |  |  |  |  |  |  |
| Mother/caregiver alone |  |  | — |  |  | — |  |
| Husband/partner/head alone |  | 1.58 | 0.88, 1.72 | 0.20 | 1.45 | 0.13, 1.23 | 0.60 |
| Husband/partner/head together |  | 1.09 | 0.01, 3.83 | 0.30 | 1.39 | 0.76, 0.98 | 0.60 |
| Another member in the household |  | 1.00 | 0.00, 1.00 | 0.021 | 1.97 | 0.45, 2.51 | 0.15 |
| Mother/caregiver and another member in the household |  | 1.92 | 1.50, -6.34 | 0.004 | 1.45 | 0.71, 3.82 | 0.20 |
| Paid care |  |  |  |  |  |  |  |
| No |  |  | — |  |  | — |  |
| Yes |  | 1.53 | 1.09, 6.27 | 0.01 | 0.10 | 1.95, 2.15 | 0.90 |
| Social development |  |  |  |  |  |  |  |
| Sex of child |  |  |  |  |  |  |  |
| Female |  |  | — |  |  | — |  |
| Male |  | 1.57 | 0.58, 1.44 | 0.30 | 1.42 | 0.42, 1.58 | 0.40 |
| Age of child (years) |  | 3.71 | 2.74, 5.43 | <0.001 | 3.36 | 2.21, 4.54 | <0.001 |
| Maternal age (years) |  |  |  |  |  |  |  |
| 15 - 24 |  |  | — |  |  | — |  |
| 25 - 24 |  | 1.04 | 0.15, 2.22 | 0.09 | 1.02 | 0.10, 1.42 | 0.80 |
| 35 - 44 |  | 1.88 | 1.49, 3.27 | 0.008 | 1.39 | 0.16, 1.94 | 0.60 |
| 45 - 49 |  | 1.28 | 0.49, 2.92 | 0.50 | 1.70 | 0.67, 2.27 | 0.40 |
| Maternal educational status |  |  |  |  |  |  |  |
| Primary not completed |  |  | — |  |  | — |  |
| At least primary |  | 1.35 | 0.43, 1.73 | 0.50 | 1.64 | 0.71, 1.44 | 0.20 |
| Maternal literacy |  |  |  |  |  |  |  |
| Cannot read and write |  |  | — |  |  | — |  |
| Can read and write |  | 1.21 | 0.55, 2.14 | 0.90 | 1.54 | 0.26, 3.35 | 0.70 |
| Mothers’ income |  |  |  |  |  |  |  |
| Not own income |  |  | — |  |  | — |  |
| Own income |  | 1.31 | 0.71, 1.34 | 0.50 | 1.59 | 0.65, 1.47 | 0.30 |
| Mothers’ marital status |  |  |  |  |  |  |  |
| Never married |  |  | — |  |  | — |  |
| Married |  | 1.50 | 0.48, 1.48 | 0.60 | 1.43 | 0.95, 2.08 | 0.70 |
| Cohabiting |  | 1.20 | 0.01, 1.61 | 0.40 | 1.69 | 0.87, 2.49 | 0.70 |
| Divorced/separated |  | 3.05 | 0.11, 9.20 | 0.30 | 1.37 | 0.77, 4.52 | 0.12 |
| Widowed |  | 1.75 | 0.91, 3.41 | 0.70 | 1.23 | 0.92, 5.39 | 0.90 |
| Maternal history of any chronic disease |  |  |  |  |  |  |  |
| No |  |  | — |  |  | — |  |
| Yes |  | 1.28 | 0.09, 2.64 | 0.07 | 1.15 | 0.01, 1.92 | 0.50 |
| Presence of father in the household |  |  |  |  |  |  |  |
| Father lived in same household |  |  | — |  |  | — |  |
| Father was not a household member and was living in the same community |  | 1.25 | 0.76, 5.26 | 0.50 | 1.60 | 0.22, 6.03 | 0.90 |
| Father was not a household member and was not living in the same community |  | 1.42 | 0.57, 4.42 | 0.30 | 1.72 | 0.64, 5.20 | 0.80 |
| Father was dead |  | 1.24 | 0.79, 5.27 | 0.15 | 1.63 | 0.59, 5.32 | 0.80 |
| Household wealth terciles |  |  |  |  |  |  |  |
| Poor |  |  | — |  |  | — |  |
| Middle |  | 1.47 | 0.73, 2.67 | 0.70 | 1.61 | 0.59, 2.81 | 0.60 |
| Rich |  | 1.44 | 0.63, 1.75 | 0.70 | 1.10 | 0.31, 2.12 | 0.90 |
| Decision to child health-seeking |  |  |  |  |  |  |  |
| Mother/caregiver alone |  |  | — |  |  | — |  |
| Husband/partner/head alone |  | 1.01 | 0.40, 1.42 | 0.90 | 1.00 | 0.36, 1.54 | 0.90 |
| Husband/partner/head together |  | 1.01 | 0.19, 1.16 | 0.09 | 1.11 | 0.11, 0.28 | 0.13 |
| Another member in the household |  | 1.29 | 0.21, 5.62 | 0.90 | 1.16 | 0.15, 7.24 | 0.81 |
| Mother/caregiver and another member in the household |  | 1.79 | 0.11, 1.54 | 0.11 | 1.27 | 0.71, 6.76 | 0.50 |
| Paid care |  |  |  |  |  |  |  |
| No |  |  | — |  |  | — |  |
| Yes |  | 2.27 | 1.65, 3.9 | 0.006 | 1.91 | 0.86, 2.68 | 0.30 |
| Emotional development |  |  |  |  |  |  |  |
| Sex of child |  |  |  |  |  |  |  |
| Female |  |  | — |  |  | — |  |
| Male |  | 0.14 | 0.02, -0.76 | 0.024 | 0.22 | 0.05, 1.00 | 0.05 |
| Age of child (years) |  | 2.73 | 2.25, 3.21 | <0.001 | 2.51 | 1.97, 3.05 | <0.001 |
| Maternal age (years) |  |  |  |  |  |  |  |
| 15 - 24 |  |  | — |  |  | — |  |
| 25 - 24 |  | 1.85 | 0.18, 3.88 | 0.07 | 0.05 | -1.87, 1.98 | 0.90 |
| 35 - 44 |  | 3.41 | 1.03, 5.80 | 0.005 | 1.49 | 0.88, 3.85 | 0.20 |
| 45 - 49 |  | 1.99 | 0.19, 3.21 | 0.30 | 1.40 | 0.46, 1.66 | 0.20 |
| Maternal educational status |  |  |  |  |  |  |  |
| Primary not completed |  |  | — |  |  | — |  |
| At least primary |  | 1.16 | 0.71, 2.02 | 0.90 | 1.25 | 0.38, 1.89 | 0.80 |
| Maternal literacy |  |  |  |  |  |  |  |
| Cannot read and write |  |  | — |  |  | — |  |
| Can read and write |  | 1.42 | 0.44, 1.60 | 0.10 | 1.13 | 0.41, 1.15 | 0.20 |
| Mothers’ income |  |  |  |  |  |  |  |
| Not own income |  |  | — |  |  | — |  |
| Own income |  | 1.09 | 0.67, 1.84 | 0.90 | 1.35 | 0.97, 1.28 | 0.70 |
| Mothers’ marital status |  |  |  |  |  |  |  |
| Never married |  |  | — |  |  | — |  |
| Married |  | 1.07 | 0.59, 3.36 | 0.90 | 1.40 | 0.25, 3.44 | 0.80 |
| Cohabiting |  | 1.55 | 0.42, 4.31 | 0.80 | 1.50 | 0.36, 4.35 | 0.80 |
| Divorced/separated |  | 2.95 | 0.71, 13.61 | 0.60 | 1.05 | 0.39, 16.49 | 0.50 |
| Widowed |  | 1.95 | 0.26, 8.16 | 0.80 | 1.43 | 0.29, 2.44 | 0.20 |
| Maternal history of any chronic disease |  |  |  |  |  |  |  |
| No |  |  | — |  |  | — |  |
| Yes |  | 1.30 | 0.06, 3.66 | 0.30 | 1.02 | 0.26, 2.22 | 0.90 |
| Presence of father in the household |  |  |  |  |  |  |  |
| Father lived in same household |  |  | — |  |  | — |  |
| Father was not a household member and was living in the same community |  | 1.04 | 0.64, 11.72 | 0.14 | 1.21 | 0.91, 15.32 | 0.30 |
| Father was not a household member and was not living in the same community |  | 1.29 | 1.30, 10.28 | 0.038 | 1.19 | 0.15, 13.94 | 0.30 |
| Father was dead |  | 1.62 | 1.56, 13.68 | <0.001 | 1.40 | 0.69, 16.5 | 0.11 |
| Household wealth terciles |  |  |  |  |  |  |  |
| Poor |  |  | — |  |  | — |  |
| Middle |  | 1.78 | 0.02, 4.58 | 0.70 | 1.14 | 0.57, 1.23 | 0.20 |
| Rich |  | 1.62 | 0.16, 4.40 | 0.70 | 1.28 | 1.66, 2.11 | 0.50 |
| Decision to child health-seeking |  |  |  |  |  |  |  |
| Mother/caregiver alone |  |  | — |  |  | — |  |
| Husband/partner/head alone |  | 1.70 | 0.69, 3.09 | 0.60 | 1.67 | 0.54, 2.88 | 0.60 |
| Husband/partner/head together |  | 1.58 | 0.59, 1.92 | 0.60 | 1.74 | 0.08, 2.55 | 0.40 |
| Another member in the household |  | 1.22 | 0.27, 4.83 | 0.30 | 1.32 | 0.19, 13.83 | 0.80 |
| Mother/caregiver and another member in the household |  | 1.22 | 1.01, 8.07 | 0.002 | 1.82 | 0.69, 7.04 | 0.30 |
| Paid care |  |  |  |  |  |  |  |
| No |  |  | — |  |  | — |  |
| Yes |  | 2.51 | 0.29, 5.31 | 0.08 | 1.02 | 0.68, 3.72 | 0.50 |
| POR = Prevalence Odds Ratio; CI = Confidence Interval. | | | | | | | |

| **Supplementary Table 2.** Factors associated with child nutrition outcomes | | | | | | | |
| --- | --- | --- | --- | --- | --- | --- | --- |
|  | **Unadjusted** | | | | **Adjusted** | | |
| **Characteristic** |  | **POR** | **95%CI** | **p-value** | **POR** | **95%CI** | **p-value** |
| **Underweight (weight-for-age z-score)** |  |  |  |  |  |  |  |
| Sex of child |  |  |  |  |  |  |  |
| Female |  |  | — |  |  | — |  |
| Male |  | 1.09 | 0.37, 1.12 | 0.50 | 1.13 | 0.45, 1.28 | 0.40 |
| Age of child (years) |  | 0.87 | 0.79, 0.97 | 0.012 | 0.86 | 0.76, 0.98 | 0.02 |
| Maternal age (years) |  |  |  |  |  |  |  |
| 15 - 24 |  |  | — |  |  | — |  |
| 25 - 24 |  | 1.09 | 0.79, 1.52 | 0.60 | 1.01 | 0.40, 2.38 | 0.90 |
| 35 - 44 |  | 1.30 | 0.69, 2.09 | 0.13 | 1.37 | 0.84, 3.11 | 0.13 |
| 45 - 49 |  | 1.29 | 0.86, 1.43 | 0.60 | 1.45 | 0.72, 1.61 | 0.40 |
| Maternal educational status |  |  |  |  |  |  |  |
| Primary not completed |  |  | — |  |  | — |  |
| At least primary |  | 1.00 | 0.31, 1.73 | 0.90 | 0.22 | 0.56, 1.11 | 0.20 |
| Maternal literacy |  |  |  |  |  |  |  |
| Cannot read and write |  |  | — |  |  | — |  |
| Can read and write |  | 1.19 | 0.58, 1.95 | 0.60 | 2.60 | 1.02, 6.61 | 0.04 |
| Mothers’ income |  |  |  |  |  |  |  |
| Not own income |  |  | — |  |  | — |  |
| Own income |  | 1.26 | 0.55, 3.02 | 0.07 | 1.24 | 0.57, 3.10 | 0.20 |
| Mothers’ marital status |  |  |  |  |  |  |  |
| Never married |  |  | — |  |  | — |  |
| Married |  | 1.04 | 0.59, 2.66 | 0.90 | 1.34 | 0.55, 4.23 | 0.50 |
| Cohabiting |  | 1.08 | 0.90, 3.73 | 0.80 | 1.11 | 0.20, 2.97 | 0.80 |
| Divorced/separated |  | 1.39 | 0.58, 4.79 | 0.50 | 1.28 | 0.73, 2.16 | 0.80 |
| Widowed |  | 1.52 | 0.77, 1.80 | 0.40 | 2.61 | 1.01, 7.21 | 0.05 |
| Maternal history of any chronic disease |  |  |  |  |  |  |  |
| No |  |  | — |  |  | — |  |
| Yes |  | 1.03 | 0.37, 3.43 | 0.90 | 1.20 | 0.28, 3.68 | 0.40 |
| Presence of father in the household |  |  |  |  |  |  |  |
| Father lived in same household |  |  | — |  |  | — |  |
| Father was not a household member and was living in the same community |  | 1.50 | 0.59, 2.59 | 0.40 | 1.49 | 0.49, 3.47 | 0.14 |
| Father was not a household member and was not living in the same community |  | 1.24 | 0.63, 1.12 | 0.60 | 1.78 | 0.99, 2.56 | 0.40 |
| Father was dead |  | 1.07 | 0.82, 1.96 | 0.90 | 1.77 | 0.03, 3.57 | 0.40 |
| Household wealth terciles |  |  |  |  |  |  |  |
| Poor |  |  | — |  |  | — |  |
| Middle |  | 1.32 | 0.94, 3.29 | 0.30 | 1.41 | 0.08, 4.27 | 0.20 |
| Rich |  | 1.47 | 0.18, 5.15 | 0.14 | 1.65 | 0.32, 5.03 | 0.06 |
| Decision to child health-seeking |  |  |  |  |  |  |  |
| Mother/caregiver alone |  |  | — |  |  | — |  |
| Husband/partner/head alone |  | 1.02 | 0.40, 4.44 | 0.90 | 1.19 | 0.66, 4.27 | 0.40 |
| Husband/partner/head together |  | 1.00 | 0.34, 5.33 | 0.90 | 1.14 | 0.51, 3.23 | 0.50 |
| Another member in the household |  | 1.32 | 0.63, 6.98 | 0.30 | 1.10 | 0.80, 3.00 | 0.90 |
| Mother/caregiver and another member in the household |  | 1.03 | 0.34, 2.27 | 0.90 | 1.06 | 0.82, 3.94 | 0.50 |
| No |  |  | — |  |  | — |  |
| Yes |  | 1.16 | 0.35, 2.66 | 0.50 | 1.04 | 0.14, 2.02 | 0.14 |
| Wasting (weight-for-length/height z-score) | | | | | | | |
| Sex of child |  |  |  |  |  |  |  |
| Female |  |  | — |  |  | — |  |
| Male |  | 1.08 | 0.26, 2.42 | 0.70 | 1.11 | 0.29, 2.52 | 0.60 |
| Age of child (years) |  | 0.84 | 0.74, 0.95 | 0.007 | 0.86 | 0.73, 1.00 | 0.05 |
| Maternal age (years) |  |  |  |  |  |  |  |
| 15 - 24 |  |  | — |  |  | — |  |
| 25 - 24 |  | 1.12 | 0.51, 3.28 | 0.60 | 1.28 | 0.77, 3.21 | 0.30 |
| 35 - 44 |  | 0.59 | 0.36, 0.97 | 0.02 | 0.51 | 0.28, 0.92 | 0.02 |
| 45 - 49 |  | 1.55 | 0.83, 1.94 | 0.40 | 1.25 | 0.83, 2.13 | 0.40 |
| Maternal educational status |  |  |  |  |  |  |  |
| Primary not completed |  |  | — |  |  | — |  |
| At least primary |  | 1.03 | 0.34, 2.41 | 0.90 | 1.21 | 0.63, 3.22 | 0.30 |
| Maternal literacy |  |  |  |  |  |  |  |
| Cannot read and write |  |  | — |  |  | — |  |
| Can read and write |  | 1.16 | 0.78, 2.09 | 0.70 | 1.84 | 0.35, 2.03 | 0.20 |
| Mothers’ income |  |  |  |  |  |  |  |
| Not own income |  |  | — |  |  | — |  |
| Own income |  | 1.23 | 0.58, 2.13 | 0.20 | 1.18 | 0.61, 2.24 | 0.40 |
| Mothers’ marital status |  |  |  |  |  |  |  |
| Never married |  |  | — |  |  | — |  |
| Married |  | 1.09 | 0.68, 2.85 | 0.80 | 1.34 | 1.79, 3.48 | 0.60 |
| Cohabiting |  | 1.07 | 0.93, 1.07 | 0.90 | 1.08 | 0.37, 1.45 | 0.90 |
| Divorced/separated |  | 1.64 | 0.08, 1.81 | 0.40 | 1.56 | 0.67, 1.94 | 0.30 |
| Widowed |  | 1.54 | 0.03, 2.11 | 0.50 | 1.26 | 0.78, 3.30 | 0.20 |
| Maternal history of any chronic disease |  |  |  |  |  |  |  |
| No |  |  | — |  |  | — |  |
| Yes |  | 1.21 | 0.28, 2.71 | 0.40 | 1.16 | 0.15, 1.87 | 0.14 |
| Presence of father in the household |  |  |  |  |  |  |  |
| Father lived in same household |  |  | — |  |  | — |  |
| Father was not a household member and was living in the same community |  | 1.65 | 0.68, 1.99 | 0.30 | 2.25 | 0.27, 4.76 | 0.08 |
| Father was not a household member and was not living in the same community |  | 1.40 | 0.67, 1.47 | 0.50 | 1.77 | 0.48, 4.03 | 0.12 |
| Father was dead |  | 1.30 | 0.79, 1.39 | 0.60 | 1.89 | 0.40, 4.18 | 0.10 |
| Household wealth terciles |  |  |  |  |  |  |  |
| Poor |  |  | — |  |  | — |  |
| Middle |  | 1.04 | 0.72, 2.79 | 0.90 | 1.07 | 0.94, 2.79 | 0.90 |
| Rich |  | 1.11 | 0.86, 2.64 | 0.80 | 1.27 | 0.13, 2.58 | 0.50 |
| Decision to child health-seeking |  |  |  |  |  |  |  |
| Mother/caregiver alone |  |  | — |  |  | — |  |
| Husband/partner/head alone |  | 1.09 | 0.61, 1.42 | 0.70 | 1.03 | 0.59, 2.29 | 0.30 |
| Husband/partner/head together |  | 1.04 | 0.45, 3.37 | 0.80 | 1.01 | 0.64, 2.30 | 0.50 |
| Another member in the household |  | 1.67 | 0.48, 2.14 | 0.60 | 1.32 | 0.36, 5.00 | 0.50 |
| Mother/caregiver and another member in the household |  | 1.26 | 0.55, 4.07 | 0.40 | 2.99 | 0.67, 6.65 | 0.11 |
| Paid care |  |  |  |  |  |  |  |
| No |  |  | — |  |  | — |  |
| Yes |  | 1.15 | 0.76, 2.47 | 0.60 | 1.04 | 0.50, 1.98 | 0.50 |
| Stunting (length/height-for-age z-score) | | | | | | | |
| Sex of child |  |  |  |  |  |  |  |
| Female |  |  | — |  |  | — |  |
| Male |  | 0.76 | 0.58, 1.00 | 0.06 | 0.66 | 0.49, -0.89 | 0.006 |
| Age of child (years) |  | 1.01 | 0.10, 1.11 | 0.90 | 1.04 | 0.16, 2.08 | 0.50 |
| Maternal age (years) |  |  |  |  |  |  |  |
| 15 - 24 |  |  | — |  |  | — |  |
| 25 - 24 |  | 1.28 | 0.05, 2.61 | 0.09 | 1.32 | 0.05, 2.68 | 0.09 |
| 35 - 44 |  | 1.16 | 0.24, 2.56 | 0.40 | 1.21 | 0.24, 2.66 | 0.40 |
| 45 - 49 |  | 1.22 | 0.39, 1.94 | 0.70 | 1.07 | 0.18, 1.94 | 0.90 |
| Maternal educational status |  |  |  |  |  |  |  |
| Primary not completed |  |  | — |  |  | — |  |
| At least primary |  | 1.02 | 0.32, 1.28 | 0.90 | 1.11 | 0.42, 2.21 | 0.50 |
| Maternal literacy |  |  |  |  |  |  |  |
| Cannot read and write |  |  | — |  |  | — |  |
| Can read and write |  | 1.28 | 0.47, 2.04 | 0.50 | 1.18 | 0.11, 1.67 | 0.08 |
| Mothers’ income |  |  |  |  |  |  |  |
| Not own income |  |  | — |  |  | — |  |
| Own income |  | 1.15 | 0.43, 2.14 | 0.30 | 1.18 | 0.50, 2.04 | 0.30 |
| Mothers’ marital status |  |  |  |  |  |  |  |
| Never married |  |  | — |  |  | — |  |
| Married |  | 1.17 | 0.80, 1.45 | 0.60 | 1.11 | 0.73, 1.96 | 0.80 |
| Cohabiting |  | 1.33 | 0.14, 1.49 | 0.40 | 1.33 | 0.36, 1.76 | 0.50 |
| Divorced/separated |  | 1.05 | 0.22, 1.81 | 0.90 | 1.22 | 0.11, 3.54 | 0.30 |
| Widowed |  | 1.12 | 0.16, 1.4 | 0.90 | 1.20 | 0.32, 2.72 | 0.12 |
| Maternal history of any chronic disease |  |  |  |  |  |  |  |
| No |  |  | — |  |  | — |  |
| Yes |  | 1.22 | 0.61, 2.18 | 0.30 | 1.22 | 0.67, 2.24 | 0.30 |
| Presence of father in the household |  |  |  |  |  |  |  |
| Father lived in same household |  |  | — |  |  | — |  |
| Father was not a household member and was living in the same community |  | 1.21 | 0.29, 2.87 | 0.70 | 1.11 | 0.99, 3.77 | 0.90 |
| Father was not a household member and was not living in the same community |  | 1.28 | 0.14, 2.59 | 0.50 | 1.73 | 0.42, 2.96 | 0.40 |
| Father was dead |  | 1.44 | 0.32, 2.44 | 0.30 | 1.16 | 0.57, 2.85 | 0.30 |
| Household wealth terciles |  |  |  |  |  |  |  |
| Poor |  |  | — |  |  | — |  |
| Middle |  | 0.52 | 0.22, 0.84 | 0.04 | 0.51 | 0.21, 0.87 | 0.040 |
| Rich |  | 0.47 | 0.19, 0.86 | 0.01 | 0.41 | 0.22, 0.71 | 0.007 |
| Decision to child health-seeking |  |  |  |  |  |  |  |
| Mother/caregiver alone |  |  | — |  |  | — |  |
| Husband/partner/head alone |  | 1.15 | 0.26, 2.55 | 0.50 | 1.00 | 0.44, 2.45 | 0.90 |
| Husband/partner/head together |  | 1.07 | 0.25, 3.39 | 0.70 | 1.02 | 0.37, 2.34 | 0.90 |
| Another member in the household |  | 1.21 | 0.42, 1.99 | 0.30 | 1.06 | 0.81, 1.69 | 0.40 |
| Mother/caregiver and another member in the household |  | 1.09 | 0.22, 1.99 | 0.40 | 1.03 | 0.77, 1.79 | 0.50 |
| Paid care |  |  |  |  |  |  |  |
| No |  |  | — |  |  | — |  |
| Yes |  | 1.65 | 1.00, 2.74 | 0.05 | 1.65 | 0.94, 2.88 | 0.07 |
| **MUAC-for-age z-scores** | | | | | | | |
| Sex of child |  |  |  |  |  |  |  |
| Female |  |  | — |  |  | — |  |
| Male |  | 1.12 | 0.13, 2.36 | 0.30 | 1.02 | 0.28, 2.24 | 0.90 |
| Age of child (years) |  | 0.76 | 0.69, 0.82 | <0.001 | 0.77 | 0.71, 0.87 | <0.001 |
| Maternal age (years) |  |  |  |  |  |  |  |
| 15 - 24 |  |  | — |  |  | — |  |
| 25 - 24 |  | 1.16 | 0.13, 2.44 | 0.30 | 1.25 | 0.07, 2.56 | 0.12 |
| 35 - 44 |  | 1.17 | 0.51, 2.17 | 0.30 | 1.03 | 0.41, 2.35 | 0.90 |
| 45 - 49 |  | 1.13 | 0.12, 1.86 | 0.80 | 1.06 | 0.99, 2.88 | 0.90 |
| Maternal educational status |  |  |  |  |  |  |  |
| Primary not completed |  |  | — |  |  | — |  |
| At least primary |  | 1.10 | 0.36, 2.17 | 0.50 | 1.00 | 0.49, 2.05 | 0.11 |
| Maternal literacy |  |  |  |  |  |  |  |
| Cannot read and write |  |  | — |  |  | — |  |
| Can read and write |  | 1.08 | 0.74, 2.58 | 0.80 | 1.66 | 0.09, 1.41 | 0.08 |
| Mothers’ income |  |  |  |  |  |  |  |
| Not own income |  |  | — |  |  | — |  |
| Own income |  | 1.16 | 0.41, 2.09 | 0.20 | 1.02 | 0.25, 2.29 | 0.90 |
| Mother's marital status |  |  |  |  |  |  |  |
| Never married |  |  | — |  |  | — |  |
| Married |  | 1.05 | 0.61, 1.59 | 0.80 | 1.05 | 0.47, 2.96 | 0.50 |
| Cohabiting |  | 1.24 | 0.97, 2.48 | 0.50 | 1.01 | 0.88, 2.89 | 0.90 |
| Divorced/separated |  | 1.21 | 0.23, 1.81 | 0.70 | 1.03 | 0.89, 2.03 | 0.40 |
| Widowed |  | 1.42 | 0.53, 1.69 | 0.50 | 1.09 | 0.78, 1.79 | 0.40 |
| Maternal history of any chronic disease |  |  |  |  |  |  |  |
| No |  |  | — |  |  | — |  |
| Yes |  | 1.09 | 0.26, 1.44 | 0.60 | 1.01 | 0.38, 1.39 | 0.90 |
| Presence of father in the household |  |  |  |  |  |  |  |
| Father lived in same household |  |  | — |  |  | — |  |
| Father was not a household member and was living in the same community |  | 1.27 | 0.24, 2.71 | 0.60 | 1.19 | 0.90, 2.28 | 0.70 |
| Father was not a household member and was not living in the same community |  | 1.24 | 0.04, 2.56 | 0.60 | 1.12 | 0.86, 1.98 | 0.50 |
| Father was dead |  | 1.58 | 0.39, 2.23 | 0.20 | 1.29 | 0.13, 2.75 | 0.30 |
| Household wealth terciles |  |  |  |  |  |  |  |
| Poor |  |  | — |  |  | — |  |
| Middle |  | 1.30 | 0.83, 2.24 | 0.30 | 1.21 | 0.75, 1.33 | 0.40 |
| Rich |  | 1.39 | 0.92, 2.14 | 0.14 | 1.36 | 0.9, 2.18 | 0.20 |
| Decision to child health-seeking |  |  |  |  |  |  |  |
| Mother/caregiver alone |  |  | — |  |  | — |  |
| Husband/partner/head alone |  | 1.20 | 0.57, 2.16 | 0.30 | 1.03 | 0.70, 2.05 | 0.08 |
| Husband/partner/head together |  | 1.14 | 0.15, 1.43 | 0.30 | 1.00 | 0.31, 1.81 | 0.90 |
| Another member in the household |  | 1.53 | 0.49, 1.42 | 0.60 | 1.29 | 0.25, 1.39 | 0.40 |
| Paid care |  |  |  |  |  |  |  |
| No |  |  | — |  |  | — |  |
| Yes |  | 1.07 | 0.50, 2.36 | 0.70 | 1.06 | 0.41, 2.53 | 0.80 |
| POR = Prevalence Odds Ratio; CI = Confidence Interval. | | | | | | | |

| **Supplementary Table 3.** Factors associated with child well-being | | | | | | | |
| --- | --- | --- | --- | --- | --- | --- | --- |
|  | **Unadjusted** | | | | **Adjusted** | | |
| **Characteristic** |  | **POR** | **95%CI** | **p-value** | **POR** | **95%CI** | **p-value** |
| **General wellbeing** |  |  |  |  |  |  |  |
| Sex of child |  |  |  |  |  |  |  |
| Female |  |  | — |  |  | — |  |
| Male |  | 1.36 | 0.86, 2.15 | 0.20 | 1.62 | 0.91, 2.89 | 0.10 |
| Age of child (years) |  | 1.00 | 0.86, 1.17 | 0.90 | 1.02 | 0.83, 1.25 | 0.80 |
| Maternal age (years) |  |  |  |  |  |  |  |
| 15 - 24 |  |  | — |  |  | — |  |
| 25 - 24 |  | 1.36 | 0.79, 2.35 | 0.30 | 1.91 | 0.93, 3.99 | 0.07 |
| 35 - 44 |  | 1.45 | 0.76, 2.8 | 0.30 | 1.94 | 0.81, 4.74 | 0.14 |
| 45 - 49 |  | - | - | - | - | - | - |
| Maternal educational status |  |  |  |  |  |  |  |
| Primary not completed |  |  | — |  |  | — |  |
| At least primary |  | 1.02 | 0.62, 1.68 | 0.90 | 1.17 | 0.64, 2.16 | 0.60 |
| Maternal literacy |  |  |  |  |  |  |  |
| Cannot read and write |  |  | — |  |  | — |  |
| Can read and write |  | 1.92 | 0.29, 2.85 | 0.90 | 1.23 | 0.04, 1.58 | 0.08 |
| Mothers’ income |  |  |  |  |  |  |  |
| Not own income |  |  | — |  |  | — |  |
| Own income |  | 1.40 | 0.87, 2.24 | 0.20 | 1.04 | 0.57, 1.88 | 0.90 |
| Mother's marital status |  |  |  |  |  |  |  |
| Never married |  |  | — |  |  | — |  |
| Married |  | 1.50 | 0.59, 4.00 | 0.40 | 2.10 | 0.47, 11.88 | 0.40 |
| Cohabiting |  | 1.53 | 0.43, 5.64 | 0.50 | 5.19 | 0.82, 40.69 | 0.09 |
| Divorced/separated |  | - | - | - | - | - | - |
| Widowed |  | 2.06 | 0.28, 18.62 | 0.50 | - | - | - |
| Maternal history of any chronic disease |  |  |  |  |  |  |  |
| No |  |  | — |  |  | — |  |
| Yes |  | 1.63 | 0.33, 2.19 | 0.20 | 1.57 | 0.24, 2.28 | 0.20 |
| Presence of father in the household |  |  |  |  |  |  |  |
| Father lived in same household |  |  | — |  |  | — |  |
| Father was not a household member and was living in the same community |  | 1.14 | 1.01, 2.60 | 0.06 | - | - | - |
| Father was not a household member and was not living in the same community |  | 1.72 | 0.14, 3.05 | 0.70 | - | - | - |
| Father was dead |  | 0.60 | 0.12, 2.58 | 0.50 | - | - | - |
| Household wealth terciles |  |  |  |  |  |  |  |
| Poor |  |  | — |  |  | — |  |
| Middle |  | 2.73 | 0.94, 9.08 | 0.07 | 3.60 | 1.28, 20.57 | 0.02 |
| Rich |  | 2.26 | 0.78, 7.49 | 0.15 | 4.58 | 1.22, 20.41 | 0.03 |
| Decision to child health-seeking |  |  |  |  |  |  |  |
| Mother/caregiver alone |  |  | — |  |  | — |  |
| Husband/partner/head alone |  | 1.05 | 0.54, 2.04 | 0.90 | 1.36 | 0.59, 3.19 | 0.50 |
| Husband/partner/head together |  | 1.05 | 0.61, 1.80 | 0.90 | 1.08 | 0.55, 2.14 | 0.80 |
| Another member in the household |  | 0.97 | 0.04, 24.83 | 0.90 | - | - | - |
| Mother/caregiver and another member in the household |  | - | - | - | - | - | - |
| Paid care |  |  |  |  |  |  |  |
| No |  |  | — |  |  | — |  |
| Yes |  | 1.23 | 0.57, 2.75 | 0.60 | 1.47 | 0.53, 4.31 | 0.50 |
| **Malaria** |  |  |  |  |  |  |  |
| Sex of child |  |  |  |  |  |  |  |
| Female |  |  | — |  |  | — |  |
| Male |  | 1.16 | 0.6, 2.24 | 0.70 | 1.24 | 0.49, 3.23 | 0.60 |
| Age of child (years) |  | 1.20 | 0.96, 1.51 | 0.11 | 1.34 | 0.95, 1.92 | 0.10 |
| Maternal age (years) |  |  |  |  |  |  |  |
| 15 - 24 |  |  | — |  |  | — |  |
| 25 - 24 |  | 1.63 | 0.28, 1.97 | 0.20 | 1.48 | 0.13, 1.71 | 0.30 |
| 35 - 44 |  | 3.67 | 1.12, 2.18 | 0.01 | 1.22 | 1.04, 2.41 | 0.03 |
| 45 - 49 |  | - | - | - | - | - | - |
| Maternal educational status |  |  |  |  |  |  |  |
| Primary not completed |  |  | — |  |  | — |  |
| At least primary |  | 1.05 | 0.52, 2.12 | 0.90 | 2.02 | 0.72, 5.90 | 0.20 |
| Maternal literacy |  |  |  |  |  |  |  |
| Cannot read and write |  |  | — |  |  | — |  |
| Can read and write |  | 1.67 | 0.35, 11.92 | 0.50 | 3.18 | 0.32, 14.74 | 0.30 |
| Mothers’ income |  |  |  |  |  |  |  |
| Not own income |  |  | — |  |  | — |  |
| Own income |  | 1.76 | 0.39, 1.98 | 0.40 | 0.73 | 0.26, 2.08 | 0.60 |
| Mother's marital status |  |  |  |  |  |  |  |
| Never married |  |  | — |  |  | — |  |
| Married |  | 1.55 | 0.12, 2.44 | 0.40 | 1.57 | 0.02, 14.26 | 0.70 |
| Cohabiting |  | 4.00 | 0.54, 9.54 | 0.20 | 3.24 | 0.06, 16.09 | 0.50 |
| Divorced/separated |  | 1.00 | 0.08, 12.21 | 0.90 | - | - | - |
| Widowed |  | 0.50 | 0.02, 7.54 | 0.60 | - | - | - |
| Maternal history of any chronic disease |  |  |  |  |  |  |  |
| No |  |  | — |  |  | — |  |
| Yes |  | 1.85 | 0.7, 4.98 | 0.20 | 2.87 | 0.77, 11.17 | 0.12 |
| Presence of father in the household |  |  |  |  |  |  |  |
| Father lived in same household |  |  | — |  |  | — |  |
| Father was not a household member and was living in the same community |  | 1.50 | 0.04, 7.09 | 0.80 | - | - | - |
| Father was not a household member and was not living in the same community |  | 0.86 | 0.14, 6.76 | 0.90 | - | - | - |
| Father was dead |  | 1.23 | 0.19, 9.96 | 0.80 | - | - | - |
| Household wealth terciles |  |  |  |  |  |  |  |
| Poor |  |  | — |  |  | — |  |
| Middle |  | 1.13 | 1.01, 2.34 | 0.05 | - | - | - |
| Rich |  | 1.21 | 0.01, 1.52 | 0.20 | - | - | - |
| Decision to child health-seeking |  |  |  |  |  |  |  |
| Mother/caregiver alone |  |  | — |  |  | — |  |
| Husband/partner/head alone |  | 2.76 | 1.09, 7.33 | 0.03 | 2.70 | 1.78, 9.84 | 0.007 |
| Husband/partner/head together |  | 1.67 | 0.29, 1.85 | 0.30 | 1.61 | 0.19, 1.89 | 0.40 |
| Another member in the household |  | - | - | - | - | - | - |
| Mother/caregiver and another member in the household |  | - | - | - | - | - | - |
| Paid care |  |  |  |  |  |  |  |
| No |  |  | — |  |  | — |  |
| Yes |  | 1.39 | 0.49, 3.88 | 0.50 | 1.41 | 0.28, 6.74 | 0.70 |
| Cold/Flu |  |  |  |  |  |  |  |
| Sex of child |  |  |  |  |  |  |  |
| Female |  |  | — |  |  | — |  |
| Male |  | 1.84 | 0.42, 1.96 | 0.60 | 1.73 | 0.29, 1.98 | 0.50 |
| Age of child (years) |  | 1.89 | 0.7, 2.11 | 0.30 | 1.78 | 0.71, 1.94 | 0.90 |
| Maternal age (years) |  |  |  |  |  |  |  |
| 15 - 24 |  |  | — |  |  | — |  |
| 25 - 24 |  | 1.20 | 0.54, 2.68 | 0.60 | 1.48 | 0.46, 4.7 | 0.50 |
| 35 - 44 |  | 1.74 | 0.66, 4.71 | 0.30 | 2.69 | 0.68, 11.53 | 0.20 |
| 45 - 49 |  | - | - | - | - | - | - |
| Maternal educational status |  |  |  |  |  |  |  |
| Primary not completed |  |  | — |  |  | — |  |
| At least primary |  | 1.03 | 0.5, 2.15 | 0.90 | 1.56 | 0.2, 1.73 | 0.30 |
| Maternal literacy |  |  |  |  |  |  |  |
| Cannot read and write |  |  | — |  |  | — |  |
| Can read and write |  | 1.44 | 0.27, 6.77 | 0.60 | 1.79 | 0.07, 7.08 | 0.80 |
| Mothers’ income |  |  |  |  |  |  |  |
| Not own income |  |  | — |  |  | — |  |
| Own income |  | 1.92 | 0.46, 2.83 | 0.80 | 1.79 | 0.29, 2.11 | 0.60 |
| Mother's marital status |  |  |  |  |  |  |  |
| Never married |  |  | — |  |  | — |  |
| Married |  | 1.36 | 0.27, 5.85 | 0.70 | 1.49 | 0.36, 8.61 | 0.20 |
| Cohabiting |  | 1.60 | 0.08, 3.94 | 0.60 | 1.86 | 0.16, 7.63 | 0.30 |
| Divorced/separated |  | 1.20 | 0.01, 2.43 | 0.20 | - | - | - |
| Widowed |  | - | - | - | - | - | - |
| Maternal history of any chronic disease |  |  |  |  |  |  |  |
| No |  |  | — |  |  | — |  |
| Yes |  | 1.54 | 0.20, 1.46 | 0.20 | 1.30 | 1.07, 2.51 | 0.03 |
| Presence of father in the household |  |  |  |  |  |  |  |
| Father lived in same household |  |  | — |  |  | — |  |
| Father was not a household member and was living in the same community |  | 1.25 | 0.17, 10.21 | 0.40 | - | - | - |
| Father was not a household member and was not living in the same community |  | 1.42 | 0.02, 2.97 | 0.40 | - | - | - |
| Father was dead |  | 1.55 | 0.30, 4.07 | 0.60 | - | - | - |
| Household wealth terciles |  |  |  |  |  |  |  |
| Poor |  |  | — |  |  | — |  |
| Middle |  | 1.62 | 0.2, 10.46 | 0.60 | 6.22 | 0.55, 9.75 | 0.13 |
| Rich |  | 1.99 | 0.12, 6.32 | 0.90 | 2.63 | 0.23, 13.63 | 0.40 |
| Decision to child health-seeking |  |  |  |  |  |  |  |
| Mother/caregiver alone |  |  | — |  |  | — |  |
| Husband/partner/head alone |  | 1.50 | 0.19, 1.98 | 0.15 | 1.20 | 1.05, 1.88 | 0.01 |
| Husband/partner/head together |  | 1.58 | 0.69, 3.75 | 0.30 | 1.41 | 0.47, 4.37 | 0.50 |
| Another member in the household |  | - | - | - | - | - | - |
| Mother/caregiver and another member in the household |  | - | - | - | - | - | - |
| Paid care |  |  |  |  |  |  |  |
| No |  |  | — |  |  | — |  |
| Yes |  | 1.67 | 0.24, 1.97 | 0.50 | 1.02 | 0.22, 5.76 | 0.90 |
| POR = Prevalence Odds Ratio; CI = Confidence Interval. | | | | | | | |
