# Supplementary material for: Association between maternal mental health and early childhood development, nutrition, and common childhood illnesses in Khwisero subcounty, Kenya
Source: PLoS One. 2025 Jan 17;20(1):e0317762. doi: 10.1371/journal.pone.0317762 (PMC11741660; doi:10.1371/journal.pone.0317762)
Supplement: S2 File — (PDF) [file pone.0317762.s002.pdf]

amref health africa

Amref Health Africa in Kenya

REF: AMREF – ESRC P679/2019 August 8, 2019

Estelle Sidze
APHRC
P.O. Box 10787-00100
Nairobi, Kenya
Tel: +25406792463
Email: esidze@aphrc.org

Dear Dr Sidze,

RESEARCH PROTOCOL: ENHANCING UNIVERSAL HEALTH COVERAGE IN KENYA THROUGH DIGITAL INNOVATION: A FINANCIAL HEALTH DIARIES EVALUATION STUDY OF M-TIBA.

Thank you for submitting your protocol to the Amref Health Africa Ethics and Scientific Review Committee (ESRC).

This is to inform you that the ESRC has reviewed and approved your protocol. Your application approval number is P679-2019. The approval period is from August 8, 2019 to August 7, 2020 and is subject to compliance with the following requirements:

a) Only approved documents (including informed consents, study instruments, advertising materials, material transfer agreement etc.) will be used.
b) All changes (amendments, deviations, violations etc.) are submitted for review and approval by Amref ESRC before implementation.
c) Death and life threatening problems and severe adverse events (SAEs) or unexpected adverse events whether related or unrelated to the study must be reported to the Amref ESRC within 72 hours of notification.
d) Any changes, anticipated or otherwise that may increase the risks or affect safety or welfare of study participants and others or affect the integrity of the research must be reported to Amref ESRC within 72 hours.
e) Clearance for export of biological specimen must be obtained from relevant government authorities for each batch of shipment/export.
f) Submission of a request for renewal of approval at least 60 days prior to expiry of the approval period. Attach a comprehensive progress report to support the renewal.
g) Submission of an executive summary report within 90 days upon completion of the study to the Amref ESRC.

Prior to commencing your study, you will be expected to obtain a research license from National Commission for Science, Technology and innovation (NACOSTI) http://oris.nacosti.go.ke/ and obtain other clearance needed.

Please do not hesitate to contact the ESRC Secretariat (esrc.kenya@amref.org) for any clarification or query.

Yours sincerely,

AMREF - ESRC 08 AUG 2019 P.O. Box 30125-00100, NAIROBI

Prof. Mohamed Karama
Chair, Amref Health Africa ESRC
CC: Samuel Muhula, Monitoring & Evaluation and Research Manager, Amref Health Africa in Kenya

Board Members: Prof R Muga | Mr P Kasimu | Mrs E Mathu | Prof P Kiama | Mrs M Kuyoh | Prof Z Qureshi | Prof J Wang'ombe | Dr D Soti | Dr G Gitahi

P O Box 30125-00100 Nairobi, Tel: +254 (0)20 699 4000, Fax: +254 (0)20 699 2531. www.amref.org

Winner of the Gates Award for Global Health
Bill & Melinda Gates Foundation
