# Supplementary material for: Association between maternal mental health and early childhood development, nutrition, and common childhood illnesses in Khwisero subcounty, Kenya
Source: PLoS One. 2025 Jan 17;20(1):e0317762. doi: 10.1371/journal.pone.0317762 (PMC11741660; doi:10.1371/journal.pone.0317762)
Supplement: Attachment — Submitted filename: Response to Reviewers.docx [file pone.0317762.s008.docx]

**Dear Dr Ammal Mokhtar Metwally	Date: 18/10/2024
Academic Editor**

**PLOS ONE**

**Subject: Point-by-point Response Letter for Submission ID [PONE-D-23-42156] - [EMID:9d1817402dbca406]**

We hope this letter finds you well. We appreciate the time and effort you and the reviewers have dedicated to assessing the second round of the above-referenced manuscript. We are grateful for the insightful comments and suggestions provided.

We have carefully considered each comment/suggestion, and we would like to assure you that all comments have been addressed meticulously and accordingly, one-by-one. Below, we provide a detailed response to the key points raised during the review process and how have addressed them.

We believe that the revisions made in response to the comments/suggestions have significantly strengthened the quality and clarity of the manuscript and supplementary materials. We are therefore confident that the changes address the concerns raised and enhance the overall contribution of our work to the field.

The main study was funded by Dutch National Postcode Lottery, the Joep Lange Institute, and the Dutch Ministry of Foreign Affairs, and EDCTP2 programme supported by the European Union and Fondation Botnar (grant number TMA2020CDF-3101-*i*-PUSH-RCT); and supported by the. The views and opinions of authors expressed herein do not necessarily state or reflect those of EDCTP and Fondation Botnar. The funders had no role in study design, data collection and analysis, decision to publish, or preparation of the manuscript.

Thank you once again for the constructive feedback, which has undeniably improved the manuscript. We look forward for your further consideration of this version of the manuscript in your esteemed journal.

Sincerely,

Amanuel Abajobir, PhD [on behalf of all authors]

**Point-by-point Responses**

**Editor:** "We note that there is identifying data in the Supporting Information file < Supplementary Table.docx >. Due to the inclusion of these potentially identifying data, we have removed this file from your file inventory. Prior to sharing human research participant data, authors should consult with an ethics committee to ensure data are shared in accordance with participant consent and all applicable local laws.

**Response:** Thank you for your feedback regarding the Supporting Information file. It is important to clarify that the Supplementary Table.docx does not contain any personally identifiable information about human research participants, as all data have been presented in an anonymized format. The intent was to ensure compliance with ethical standards while providing relevant information. If necessary, we are happy to provide further details or make adjustments to the file to align with your guidelines. Please let us know how best to proceed.

***Reviewer 1***

Reviewer #1: Thank you for submitting this important work. Please consider the following recommendations:
1. Please indicate the research question for the parent study so readers can evaluate the relationship between the studies.

**Response:** As indicated in this published protocol (Abajobir A, de Groot R, Wainaina C, Njeri A, Maina D, Njoki S, Mbaya N, Donfouet HPP, Pradhan M, Janssens W, Sidze EM. The impact of i-PUSH on maternal and child health care utilization, health outcomes, and financial protection: study protocol for a cluster randomized controlled trial based on financial and health diaries data. Trials. 2021 Sep 15;22(1):629. doi: 10.1186/s13063-021-05598-7), the main/parent study aims to evaluate the impact of *i*-PUSH intervention on maternal and child health care services utilization, women’s health including their knowledge, behavior, and uptake of respective services, as well as women’s empowerment and financial protection. It also aims to evaluate the impact of the LEAP training tool on empowering and enhancing community health volunteers’ health literacy and to evaluate the impact of the M-TIBA health wallet on savings for health and health insurance uptake. Because of space/word limitations as per requirements of the journal, we cited this reference from outset so readers can refer to it. We also encourage readers for other publications on other relevant health outcomes here: <https://academic.oup.com/heapol/advance-article/doi/10.1093/heapol/czae079/7741192?searchresult=1>; <https://pubmed.ncbi.nlm.nih.gov/37448326/>.

2. If that question is related at all to the research question of this study, then you should explore participation in the intervention group as a potential confounder.

**Response:** As stated above, the research questions of the parent study were not directly related to the current research question, and thus, the intervention was not considered as a potential confounder in our analyses.

3. Demographic issues: participation by women under 18 is unusual. Indicate how many minors participated. Also, how many women serving as caretakers but not related to the child participated in this study? Could that be confounding the association?

**Response:**

- number of women under 18 (15 – 17) years was 9, and participants who belonged to this age category have already been included in the age category of 15-24 years (see Supplementary Tables). We have also included this number in the Methods section on page 9.
- Only a few mothers alternate the caregiving role with non-biological caretakers (mostly grandparents) in a few households. So, it was difficult to delineate their role and include them separately in the analysis, as the number was so low (less than 10) and childcare giving roles overlap. This information has been provided in the Methods section on page 6.

4. Were the ECD scores assessed by age group? Otherwise, it is normal for older children to show more advanced development than younger children. It doesn't make sense to analyze such a large age range (8-60 months) together using the same ECD measures for all of them. What you want to know is, is each child developmentally appropriate for their age. I don't see this info in the supplemental information.

**Response:** The tool used to assess ECD was validated across the 8-60 month age range, and scores were not stratified by age. We agree that older children typically show more advanced development. To account for this, we adjusted for age in the analysis to correct for potential variation in development across age groups.

5. Please include tables in the supplementary materials for all results reported for which you note, "Table not shown."

**Response:** Thank you. These Tables and their respective data have now been included in the Supplementary.

6. Methods: Typically, the effect measure for cross-sectional studies is a POR. It would be better / more useful to report and interpret the effect measures rather than simply report the beta coefficients.

Response: We have replaced the beta coefficients with Prevalence Odds Ratios (POR) and updated the relevant tables (see Supplementary Tables 1–3).

7. Results section: In the third line, you say "for both groups." But this study has just one group.

**Response:** This was a typographical error, which has been corrected.

8. Please report the possible ranges for all scores reported (perhaps when you describe the measures in the Methods section) because the reader cannot evaluate whether reported means are high or low, or otherwise clinically important.

**Response:** The score ranges have already been included in Table 1 and described in the Methods section (see pages 6–8).

9. Please rewrite the first paragraph of the Discussion to restate the main findings of the study and its significance. Then move into comparisons to other studies.

**Response:** The first paragraph of the Discussion has been added to summarize the key findings and their significance before delving into comparisons with other research (page 15).

10. I'd like to see more discussion about why there were unexpected findings.

**Response:** We have expanded the discussion to address potential reasons for the unexpected findings, including unmeasured confounders, the influence of contextual factors such as socio-economic disparities, or differences in healthcare access across regions (page 16).

11. Please check the manuscript for correct English grammar, e.g., see the first sentence under "Confounders."

**Response:** The manuscript has been thoroughly reviewed for grammar, and the first sentence under "Confounders" has been revised for clarity and correctness.

***Reviewer 2***

Reviewer #2: Overall a sound and thorough research paper with robust statistical conclusions.
Additionally, the history of childhood vaccinations recommended for first 2 years of life and maternal tetanus vaccinations that are given during or before pregnancy could have also been included as covariates.

**Response:** We considered including vaccination history as covariates. However, due to substantial missing data on these variables at baseline survey, it was not possible to include them in the final analysis without compromising the validity of the results.

Moreover, since this is a cross sectional study, instead of reporting B coefficients of logistic regression, Prevalence Odds ratios (POR) along with their confidence intervals and p values should be reported.

**Response:** We rerun the analyses and changed the coefficients to POR (see Tables 2 and Supplementary Tables 1–3).
